# The Dysfunctional Right Ventricle in Dilated Cardiomyopathies: Looking from the Right Point of View

**DOI:** 10.3390/jcdd9100359

**Published:** 2022-10-19

**Authors:** Maria L. Iovănescu, Diana R. Florescu, Andreea S. Marcu, Ionuț Donoiu, Sebastian Militaru, Cristina Florescu, Octavian Istrătoaie, Constantin Militaru

**Affiliations:** 1 Department of Cardiology, University of Medicine and Pharmacy of Craiova, 200349 Craiova, Romania; 2 Clinical Emergency County Hospital of Craiova, 200642 Craiova, Romania; 3 Department of Physiology, University of Medicine and Pharmacy of Craiova, 200349 Craiova, Romania; 4 Filantropia Clinical Hospital, 200516 Craiova, Romania

**Keywords:** right ventricular dysfunction, dilated cardiomyopathy, echocardiography, multi-modality imaging

## Abstract

Dilated cardiomyopathies (DCMs) are a heterogenous group of primary myocardial diseases, representing one of the leading causes of heart failure, and the main indication for heart transplantation. While the degree of left ventricular dilation and dysfunction are two key determinants of adverse outcomes in DCM patients, right ventricular (RV) remodeling and dysfunction further negatively influence patient prognosis. Consequently, RV functional assessment and diagnosing RV involvement by using an integrative approach based on multimodality imaging is of paramount importance in the evaluation of DCM patients and provides incremental prognostic and therapeutic information. Transthoracic echocardiography remains the first-line imaging modality used for the assessment of the RV, and newer techniques such as speckle-tracking and three-dimensional echocardiography significantly improve its diagnostic and prognostic accuracy. Nonetheless, cardiac magnetic resonance (CMR) is considered the gold standard imaging modality for the evaluation of RV size and function, and all DCM patients should be evaluated by CMR at least once. Accordingly, this review provides a comprehensive overview of the anatomy and function of the RV, and the pathophysiology, diagnosis, and prognostic value of RV dysfunction in DCM patients, based on traditional and novel imaging techniques.

## 1. Introduction

Dilated cardiomyopathies (DCMs) are primary myocardial disorders characterized by left ventricular or biventricular dilation and systolic dysfunction in the absence of either abnormal pressure or loading conditions or coronary artery disease [1,2]. DCM should not be regarded as a single disease entity, but rather as the final common response of the myocardium to a large spectrum of genetic and non-genetic insults. Since it is one of the leading causes of heart failure (HF), and the most frequent indication for heart transplantation, in the past few decades, extensive progress has been made in the development and recognition of newer diagnostic and therapeutic strategies for the management of patients with DCM. The degrees of left ventricular (LV) dilation and systolic dysfunction have been demonstrated as two key determinants of adverse outcomes in this population, and LV reverse remodeling has become the cornerstone of the treatment of DCM patients [3]. However, adverse right ventricular (RV) remodeling and RV dysfunction both at diagnosis and during follow-up yield important prognostic implications and have accordingly become the focus of many authors in recent years [4,5]. Therefore, this review aims to provide an overview of the RV involvement in DCM patients, as well as the prognostic significance of RV dysfunction assessed by multi-modality cardiac imaging.

## 2. Anatomy and Function of the Normal Right Ventricle

**Figure 1:**
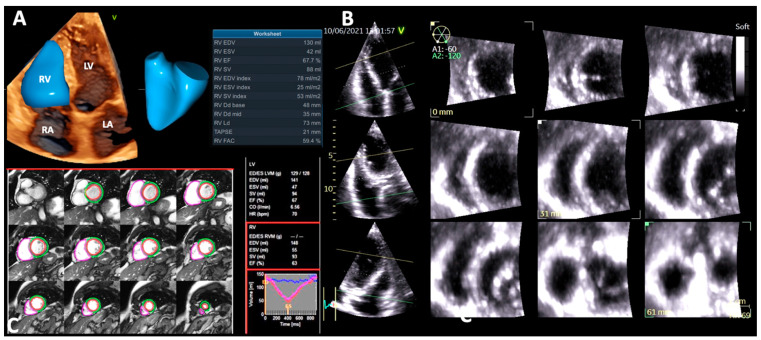
Right ventricular geometry and function in a normal subject assessed by three-dimensional echocardiography. (**A**) Right ventricular volumes and ejection fraction. (**B**) Multi-slice view of the right ventricle from the three-dimensional echocardiography data set showing the crescent shape in cross-section and triangular shape when viewed from the side. (**C**) Right ventricular geometry and function in a normal subject by cardiac magnetic resonance. Abbreviations: CO, cardiac output; Dd, diastolic diameter; EDV, end-diastolic volume; EF, ejection fraction; ESV, end-systolic volume; FAC, fractional area change; HR, heart rate; LA, left atrium; LD, longitudinal diameter; LV, left ventricle; LVM, left ventricular mass; RA, right atrium; RV, right ventricle; RVM, right ventricular mass; SV, stroke volume; TAPSE, tricuspid annulus plane systolic excursion.

Several anatomic features distinguish the RV from the LV. Being the most anteriorly situated cardiac chamber, the RV has a complex shape, which partially explains the more difficult imaging evaluation of the RV compared to the LV. With a crescent shape when viewed in cross-section, and triangular when looking from the side, the RV consists of three components: (1) the inlet, containing the tricuspid valve, chordae tendinae, and the papillary muscles; (2) the trabeculated apical myocardium; and (3) the infundibular or conal region, which accommodates the outflow tract and supports the pulmonary valve (Figure 1) [6,7]. Different from the LV, the inlet and outlet components are separated by the crista supraventricularis, a muscular crest which continues as a parietal band at the level of the free wall, and as the septomarginal band across the interventricular septum (IVS) [8]. This muscular bridge is involved in the contraction of the tricuspid annulus (TA) and in pulling the free wall of the RV towards the IVS [8,9]. The RV acts mainly as a volumetric pump. Since the pulmonary vascular resistance (PVR) is about one-sixth compared to the systemic resistance, the RV mass is much smaller than the LV mass, and the normal thickness of the RV free wall does not exceed 5 mm [10]. Furthermore, the RV has a different myoarchitecture, lacking a well-defined mid-layer. It comprises a superficial layer, with predominantly circumferentially oriented myocardial fibers, parallel to the atrioventricular groove, extending from one ventricle to another, and a preponderant subendocardial layer, which mostly has base-to-apex longitudinally oriented myocytes that are continuous with septal fibers.

The distinct hemodynamic environment (low vascular resistance, increased pulmonary artery distensibility) explains the organized fashion in which the RV contracts. The contraction of the RV resembles a peristaltic motion, with the inlet and apical regions contracting first, and the conal one 20 to 50 ms later [11]. The flow patterns within the RV are smoother compared to the vortex-like flow organization of the LV [10,12]. The contraction of the longitudinal fibers is responsible for the drawing of the TA towards the apex and shortening of the RV long axis. This longitudinal shortening accounts for a significant percentage of RV contraction and remains important in pressure-overload states [13]. Contraction of the circumferential fibers produces a bellows effect, with an inward motion of the RV free wall. Finally, the contraction of the LV drags the RV free wall through the IVS at the points of attachment, contributing to generating more than 20% of the RV stroke volume (SV) [14]. Thus, these non-longitudinal motion components play an important role in the normal functioning of RV [15].

## 3. Right Ventricular Dysfunction

The RV performance is the result of the interaction between contractility, afterload, and preload. Through the concept of ventricular interdependence, the size, shape, and compliance of one ventricle affect the other. Consequently, RV function is directly influenced by the systolic ventricular interdependence, through the IVS. The diastolic ventricular interdependence is mostly related to the degree of pericardial constraint [16]. The two main conditions that impact and dictate RV function are hemodynamic overload, both in terms of pressure and/or volume overload, and intrinsic RV contractile dysfunction [10]. These mechanisms frequently coexist. The prevalence of RV dysfunction in the setting of DCM has been reported to be 34–65% [4,17,18]. The adverse RV remodeling may result from any of the aforementioned conditions: pressure overload (pulmonary arterial hypertension, PAH), volume overload (significant tricuspid regurgitation, TR), primary myocardial disease, or a combination of them [10,19]. For example, in DCM patients affected by hereditary muscular dystrophies [20], RV longstanding volume overload and dysfunction [21] can develop in the context of a direct atrial myopathic process that contributes to the development of AF, and consecutive atrial secondary TR [22,23].

On one end, the development of PAH secondary to left-sided cardiac disease has a complex pathophysiology that involves the interplay between the LV, the left atrium (LA), and the mitral valve (MV) apparatus [19]. The enlargement and increase in the sphericity of the LV are responsible for the occurrence of functional mitral regurgitation (MR), which combined with both impaired systolic and diastolic LV function usually leads to adverse LA remodeling and loss of the LA reservoir, conduit, and pump functions, as well as decrease in the barrier-like role of the LA between the pulmonary circulation and the LV [24]. A stiff and non-compliant LA further contributes to the backward transmission of the increased hydrostatic pressure to the pulmonary vascular bed. The persistently elevated hydrostatic pressure then induces pulmonary vascular remodeling, and finally leads to an increase in the PVR [25,26,27]. Nonetheless, isolated post-capillary pulmonary hypertension is also possible [28]. However, irrespective of the etiology of PAH, in the setting of chronic pressure overload, the RV initially passes through a homeometric phase, with preserved volumes and function, and a compensatory increase in wall thickness to reduce the wall tension. If left untreated, the heterometric phase of RV maladaptive remodeling occurs, with progressive RV dilation and dysfunction [10,29,30]. Nevertheless, the diastolic function of the RV can also be impaired.

On the other end, the myopathic involvement of the RV in DCM is multifactorial and depends on the underlying etiology. Thirty to forty percent of patients with DCM have biventricular involvement, with the same myopathic process affecting both ventricles. One strong predictor of RV dysfunction is a reduced LVEF [31]. Furthermore, due to the systolic ventricular interdependence, the impaired systolic function of the IVS in DCM patients can lead to an important reduction in RV SV [16,31]. The diastolic ventricular interdependence plays a secondary role in determining RV dysfunction in the setting of LV overload [32].

## 4. Multi-Modality Imaging Evaluation of the Right Ventricle

RV function has been shown to significantly impact patient prognosis in several cardiac conditions [4,31,33,34,35,36], thus highlighting the importance of its accurate functional evaluation by the use of multi-modality imaging consisting of echocardiography, cardiac magnetic resonance (CMR), and cardiac computed tomography (CCT) in a complementary fashion [37,38].

### 4.1. Echocardiography

Although not considered the gold-standard technique for the evaluation of the RV due to multiple considerations (position, complex geometry, difficult endocardial tracing, load-dependency), transthoracic echocardiography (TTE) remains the first-line imaging modality used for the assessment of the RV due to its wide availability and cost-efficiency. The functional evaluation of the RV by TTE is usually based on two-dimensional echocardiography (2DE). However, advanced echocardiographic techniques, such as speckle-tracking echocardiography (STE) and three-dimensional echocardiography (3DE), have become mandatory for the accurate quantification of the RV function [38,39,40,41]. Table 1 provides an overview of the advantages, disadvantages, and prognostic value of the main echocardiographic parameters used for the assessment of RV systolic function.

#### 4.1.1. Tricuspid Annulus Plane Systolic Excursion by M-Mode Echocardiography

**Figure 2:**
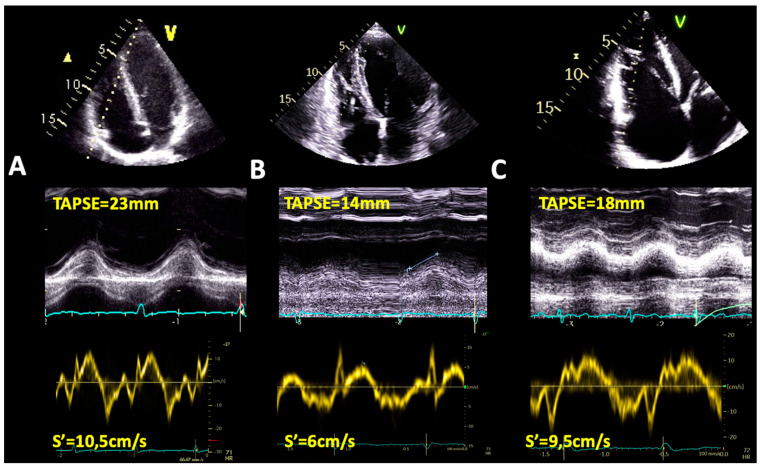
Right ventricular function assessed by M–mode (TAPSE), upper panel, and tissue Doppler imaging (S’ wave velocity), lower panel in (**A**) a normal subject, (**B**) a patient with dilated cardiomyopathy and normal right ventricular size and longitudinal dysfunction, and (**C**) a patient with dilated cardiomyopathy and biventricular involvement, yet normal right ventricular longitudinal function. Abbreviations as in Figure 1.

TA plane systolic excursion (TAPSE) measurement by M-mode echocardiography evaluates the systolic motion of the TA towards the RV apex at the level of the lateral TA (Figure 2) [42]. However, it has significant limitations (i.e., it assesses the longitudinal systolic function of the RV at the level of one segment and cannot be utilized to evaluate the global RV systolic performance; it is highly dependent on load and angle, leading to over- or underestimation of RV function) [43]. Increased afterload is often associated with a reduced TAPSE, without an actual decrease in RV longitudinal systolic function [44]. An increased RV preload can lead to overestimation of the RV function when its quantification is based on this sole parameter [45]. However, it remains an accessible parameter that has adequate reproducibility.

A TAPSE < 16 mm is considered suggestive of RV systolic dysfunction [42]. The prognostic role of TAPSE in patients with non-ischemic DCM has been extensively studied. Venner et al. found that patients with DCM and preserved RV function had significantly higher survival rates compared to those with RV systolic dysfunction defined by a TAPSE < 15 mm, irrespective of the degree of LV dysfunction. Major adverse cardiac event-free survival rates at 1 and 2 years were 64% and 55%, respectively, compared with the patients which had preserved RV function, whose survival rates were 87% and 79%, respectively. A reduced TAPSE appeared as an independent predictor for major cardiac events [17]. Ghio et al. demonstrated that in DCM patients a TAPSE ≤ 14 mm emerged as an independent predictor of death or urgent cardiac transplantation [46]. Furthermore, Ishiwata et al. found TAPSE to be independently associated with the primary composite outcome consisting of left ventricular assist device (LVAD) implantation or all-cause death in patients with non-ischemic DCM [47]. The prognostic role of the RV has also been assessed in acute HF patients with different EF ranges by Roger Hullin et al., who demonstrated that an increase in TAPSE secondary to decongestive HF treatment was associated with a lower incidence of the combined outcome, irrespective of the LVEF [48]. The prognostic significance of RV systolic dysfunction defined as TAPSE < 17 mm was also confirmed by Dziewiecka et al. in a large study on 545 DCM patients, however not irrespective of LVEF [49]. In contrast, Kawata et al. did not find any association between TAPSE reduction and the outcome consisting of LVAD implantation or cardiac death within one year when compared to other RV functional parameters in DCM patients with advanced HF [50].

#### 4.1.2. S’ Wave Velocity by Tissue Doppler Imaging

The systolic velocity of the lateral TA by tissue Doppler imaging (TDI) is another measurement of RV longitudinal function (Figure 2) [43]. Furthermore, similar to TAPSE, despite it being angle-dependent and not able to assess global RV systolic function, it is easily obtainable and has been used for the stratification of patient prognosis. An S’ wave < 9.5 cm/sec defines RV systolic dysfunction [49]. De Groote et al. showed that although RV ejection fraction (RVEF) had the highest accuracy for predicting patient survival, the measurement of both RVEF and S’ wave velocity provided increased prognostic value compared to the sole use of RVEF [51]. In another study on patients with HF, Dokainish et al. demonstrated the independent association of the S’ wave velocity with the outcome [52]. However, a small retrospective study on DCM patients with advanced HF did not find any correlation between a reduced S’ wave velocity and the chosen outcome [50].

#### 4.1.3. Right Ventricular Myocardial Performance Index by Tissue Doppler Imaging/Pulsed Doppler

The RV myocardial performance index (RV MPI) or Tei index is a unitless parameter which provides information about global systolic and diastolic RV function. It can be measured either with TDI or pulsed Doppler (PW) by dividing the total isovolumic time (isovolumic contraction time, ICT, plus isovolumic relaxation time, IRT) to the ejection time (ET). The TDI method has the advantage of simultaneously recording time intervals from the same cardiac cycle. Since this measurement is based only on time intervals, it surpasses the limitations of RV shape and geometry. However, it is less useful in irregular heart rhythms. Proposed cut-offs for an abnormal RV MPI are >0.43 (PW) and >0.54 (TDI) [53]. Impaired RV global function is defined by a high RV MPI. In case of systolic dysfunction, ICT is prolonged and ET is shortened, while a prolonged IRT is encountered in both systolic and diastolic dysfunction. Some authors suggest there is a correlation between TDI-derived RV MPI and RV ejection fraction and RV fractional area change [54]. The prognostic role of RV MPI has been studied in different cohorts with HF with reduced ejection fraction (HFrEF). In a study by Enrico Vizzardi et al. on patients with moderate HF, an RV MPI > 0.38 along with a reduced TAPSE predicted the prognosis at 5-year follow-up [55]. Field et al. demonstrated that RV dysfunction, defined as an increased RV MPI value, was associated with adverse outcomes in a population of advanced HF patients referred to cardiac resynchronization therapy (CRT). Each 0.1-unit increase in RV MPI was associated with a 16% increased risk of MACEs (defined as death of all-cause, cardiac transplantation, or ventricular assist device placement) [56]. Although, to the best of our knowledge, no studies have specifically addressed the prognostic value of Tei index in DCM, it is an easily obtainable parameter which requires little experience in the evaluation of the RV.

#### 4.1.4. Right Ventricular Fractional Area Change by Two-Dimensional Echocardiography

**Figure 3:**
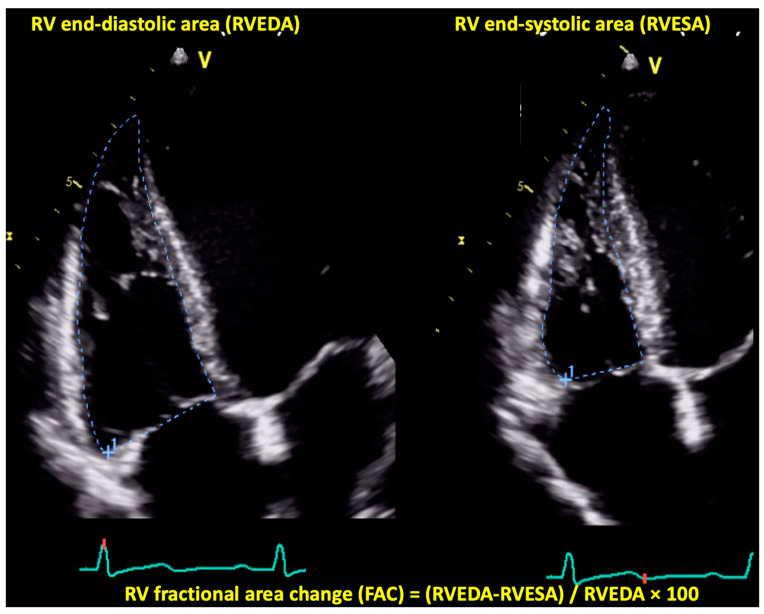
Right ventricular fractional area change calculation by two-dimensional echocardiography. Abbreviations: RVEDA, right ventricular end-diastolic area; RVESA, right ventricular end-systolic area; other abbreviations as in Figure 1.

RV fractional area change (FAC) is obtained by measuring RV end-diastolic and end-systolic areas after manually tracing the RV endocardial borders in the RV-focused apical 4 chamber (A4C) view (Figure 3) [53]. RV FAC evaluates both the longitudinal and the radial shortening of the RV. Despite being relatively easy to acquire, suboptimal image quality and the numerous RV trabeculations can sometimes lead to inaccurate measurements. Furthermore, RV FAC measurement neglects the contribution of the RV outflow to the RV global contractile function [57]. An RV FAC < 35% defines RV systolic dysfunction [42]. In DCM patients, a reduced RV FAC, with a cut-off value of 26.7%, was associated with increased risk of LVAD implantation or cardiac death within one year [50]. In another large study on 512 patients with DCM by Merlo et al., RV dysfunction, defined as an RV FAC < 35%, was independently associated with the primary outcome of death or heart transplantation, and its independent predictive value was maintained over time, suggesting an additive prognostic value during long-term follow-up [5]. Finally, the results of the study by Ishiwata et al. were consistent with previous findings demonstrating that a reduced RV FAC is an independent predictor of LVAD implantation and all-cause death [52].

#### 4.1.5. Right Ventricular Strain by Speckle-Tracking Echocardiography

**Figure 4:**
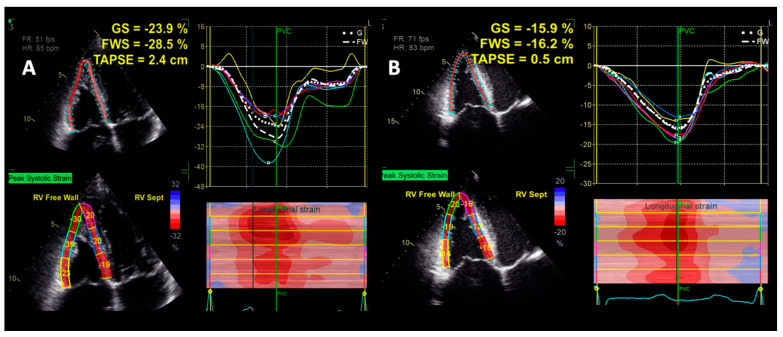
Right ventricular functional assessment by speckle–tracking echocardiography in a patient with (**A**) normal right ventricular longitudinal function, and (**B**) right ventricular longitudinal dysfunction. Abbreviations: FWS, free wall strain; GS, global strain; other abbreviations as in Figure 1.

The systolic performance of the RV can be evaluated with the use of myocardial deformation imaging techniques. The current recommendation in clinical practice is to evaluate the RV free wall longitudinal strain (FWLS) in the RV-focused A4C view by STE (Figure 4) [53,58]. The RV global longitudinal strain (GLS) can, however, be measured by including the IVS in the analysis, with adequate inter- and intra-observer variability [59,60]. The RV FWLS has incremental prognostic value compared with the conventional indices of RV systolic function in patients with various cardiac diseases [35,61,62,63,64], including DCM. Vijiiac et al. demonstrated the prognostic significance of RV dysfunction in DCM patients, as patients with more impaired RVFWLS, RVGLS, and RVEF had higher risk of event occurrence, defined as cardiac death, nonfatal cardiac arrest, or acute worsening of HF requiring hospitalization [65]. Conversely, Seo et al. showed that only RV FWLS (and not RV GLS), with a cut-off value of −16.5%, was an independent predictor of the outcome in patients with DCM [66]. Garcia-Martin et al. found that RV GLS is a superior determinant of HF decompensation compared with RV FWLS in patients with left heart disease [67]. Ishiwata et al. showed that a reduced RV GLS, especially when combined with a reduced RV FAC, is a valuable predictor of adverse events in patients with DCM [52]. However, the exact superiority of either RV FWLS or RV GLS over the other in predicting patient prognosis remains to be demonstrated.

#### 4.1.6. Right Ventricular Ejection Fraction by Three-Dimensional Echocardiography

**Figure 5:**
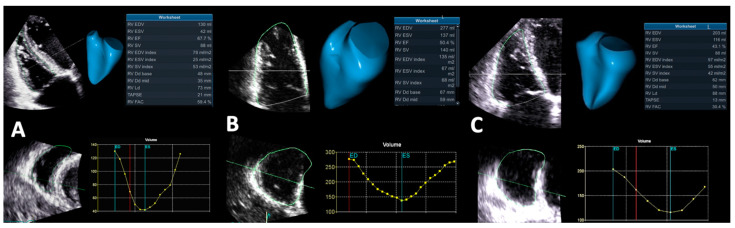
Right ventricular volumes and ejection fraction calculation by three-dimensional echocardiography in (**A**) a normal subject, (**B**) a patient with right ventricular dilation and preserved systolic function, and (**C**) a patient with right ventricular dilation and dysfunction. Abbreviations as in Figure 1.

The aforementioned limitations of classical RV function parameters assessed by 2DE (i.e., the geometric assumptions involved in their quantification, and the fact that they do not take into account the contribution of all RV components to the RV contraction) can be overcome by the use of 3DE [57]. Furthermore, RVEF by 3DE is the only RV functional echocardiographic parameter accurate enough in assessing RV function after cardiac surgery (Table 1) [53]. Still, RV assessment by 3DE is challenging in the presence of suboptimal acoustic windows which make endocardial border detection burdensome, irregular heart rhythms, or abnormal septal motion. It is recommended to use full volume data sets and multi-beat acquisition. The precise reconstruction of RV geometry and obtainment of reliable RV volumes and RVEF calculations rely on optimal spatial and temporal resolution [68,69], and involve the use of a dedicated software for the 3DE RV analysis (Figure 5).

A 3DE RVEF < 45% is consistent with RV dysfunction [53]. Despite the fact that 3DE underestimates RV volumes and EF compared to CMR [70], 3DE RV volumes and EF have been validated against CMR [71]. Leibundgut et al. found a small difference in RV volumes calculations and no significant difference in RVEF measurement between 3DE and CMR [72]. Accordingly, the functional evaluation of the RV by 3DE is a valuable alternative to CMR. In the study by Vijiiac et al. 3DE RVEF, with a cut-off value of 43.4%, predicted the outcome in DCM patients and remained independently associated with patients’ mortality even after correcting for other echocardiographic confounders [65]. D’Andrea et al. demonstrated the correlation between 3DE RVEF and VO2 peak %, as well as its independent association with the functional capacity of patients with DCM patients [73]. The prognostic value of 3DE RVEF is furthermore confirmed in several studies on patients with various cardiovascular diseases, including DCM [36,74,75].

All the aforementioned echocardiographic parameters, each with several advantages and limitations, have proven useful in diagnosing RV dysfunction in DCM patients, especially when the evaluation follows a multiparametric approach. Furthermore, recent studies suggest that the presence and pattern of RV dysfunction influence the response to HFrEF therapies. Yanis Bouali et al. evaluated two HFrEF phenotypes undergoing treatment with sacubitril/valsartan (S/V). Among the dissimilarities between the phenotypes, TAPSE (16 ± 4 mm vs. 19 ± 4 mm), RVFWLS (−19 ± 5% vs. −21 ± 4%), and RVFAC (31 ± 9% vs. 38 ± 9%) were found to be significantly lower at the initiation of therapy compared to follow-up. The phenotype with more significant RV failure at baseline had worse prognosis during the treatment period. However, the evolution of RV function was characterized by a significant improvement in TAPSE, RVFAC, and RVFWLS in both groups. Moreover, the improvement in TAPSE was correlated with both LVEF and LV GLS. These findings support the belief that there is a connection between LV and RV reverse remodeling in patients undergoing HF therapies which have been mainly attributed to LV dysfunction [76]. A systematic review and meta-analysis on the effect of S/V on the RV function in patients with HFrEF showed that RV systolic performance, evaluated by TAPSE and S’ wave velocity, improves after S/V treatment initiation [77]. Accordingly, not only LV function parameters, but also easily obtainable indices of RV function, such as TAPSE, S’ wave velocity, and RVFWLS should be measured when evaluating the response to treatment in HFrEF patients.

Finally, dimensional and functional RV parameters obtained by echocardiography are helpful in differentiating physiological RV remodeling found in athlete’s heart from the pathological RV remodeling encountered in conditions such as arrhythmogenic right ventricular cardiomyopathy (ARVC). Although the RV dimensional remodeling has a dissimilar pattern in the two conditions [78], with the dynamic exercise-induced remodeling characteristic of athlete’s heart being represented by a moderate increase in RV main body and a mild increase in RVOT size, and important RVOT dilation being found in ARVC [79], RV function parameters are much more effective in distinguishing between the two. RV FAC, S’ wave velocity, and RVFWLS are usually normal in athlete’s heart compared to ARVC [80,81]. However, the studies on RV strain show contrasting results, some authors describing supernormal values, and others lower values (generally confined to the basal segment) in athlete’s heart compared to ARVC [82]. Furthermore, a lower reference value has recently been proposed for RVFAC in athletes (<32%) compared to the general population [71]. Nonetheless, the majority of these findings reinforce the importance of a multiparametric RV evaluation when differentiating physiological from pathological RV remodeling.

#### 4.1.7. Right Ventriculo-Arterial Coupling Evaluation Using Invasive Versus Non-Invasive Parameters

Pressure–volume loops (PVL) derived from right heart catheterization (RHC) are considered the gold-standard methods for the evaluation of RV function in relation to the afterload. The relationship between RV contractility and afterload is described as right ventriculo-arterial coupling (RVAC), and is defined as the ratio between end-systolic elastance (Ees) and arterial elastance (Ea). A normal Ees/Ea reflects an optimal balance between RV mechanical work and oxygen consumption [83,84]. In left-sided heart failure with secondary PH, once maladaptive RV remodeling and dysfunction occur, the RVAC is also impaired. However, RVAC by invasively derived PVL by RHC are not routinely performed. As such, echocardiographic surrogates of RVAC have been proposed, including TAPSE/sPAP (systolic pulmonary artery pressure), RVFAC/sPAP, RVLS/sPAP, RV end-systolic volume/stroke volume (ESV/SV), and RVEF/sPAP. TAPSE/sPAP has been found to be a valuable non-invasive measure of RVAC in patients with PH [85]. Despite the fact that the prognostic significance of the previously mentioned non-invasive RVAC surrogate parameters has been extensively evaluated in patients with left-sided HF and secondary PH-related RV dysfunction, the results are variable and sometimes conflicting. In a prospective observational study, Bosch et al. found that TAPSE/sPAP and RVLS/sPAP are associated with the composite outcome of death and HF hospitalization after a mean follow-up of 2 years [86]. Furthermore, a reduced TAPSE/sPAP predicted CRT non-responders, but it did not predict survival at four years in a study on HF patients by Braganca et al. [87]. TAPSE/sPAP was associated with lower survival and the need for LVAD implantation or cardiac transplant in different HFrEF cohorts [18,88,89,90,91]. In a study on DCM patients, five non-invasive echocardiographic surrogates of RVAC were evaluated as potential rehospitalization predictors (TAPSE/sPAP, RVLS/sPAP, RVFWLS/sPAP, 3D RVEF/sPAP, 3D RV SV/ESV). Although all five surrogates were more impaired in DCM patients requiring rehospitalization for HF exacerbation, only RVFWLS/sPAP and 3D RVEF/sPAP remained independently associated with the composite outcome of hospitalization for decompensated HF and death [92]. Accordingly, non-invasive RVAC has significant prognostic value and should be used for patient risk stratification.

### 4.2. Cardiac Magnetic Resonance

**Figure 6:**
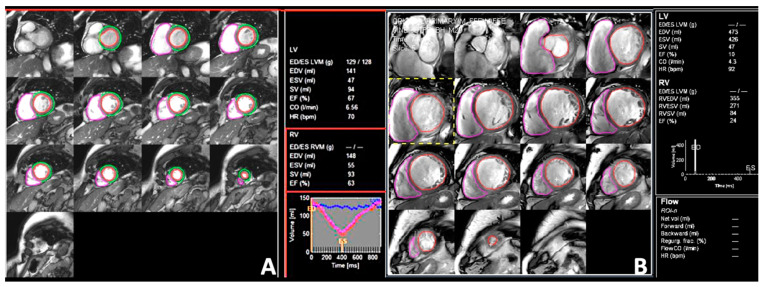
Right ventricular volumes and ejection fraction calculated by cardiac magnetic resonance in (**A**) a normal subject and (**B**) a patient with dilated cardiomyopathy. Abbreviations as in Figure 1.

The assessment of RV systolic function in patients with DCM started more than 2 decades ago, with the evaluation of RVEF by invasive techniques with limited clinical applicability, such as thermodilution or contrast ventriculography [93,94,95]. Nowadays, CMR is considered the gold-standard imaging modality for the evaluation of RV volumes and EF (Figure 6), and due to its non-invasive character and high accuracy, its applications have been widely extended over time. Besides RV volumes and EF assessment, CMR can also differentiate physiological from pathological RV remodeling thanks to its ability to detect tissue abnormalities (fat infiltration, late gadolinium enhancement) [4,79]. CMR evaluation in DCM patients has a class IIa indication for the exclusion of an ischemic etiology [96], and might indicate the etiology of cardiac dysfunction by the use of myocardial tissue characterization techniques [94]. Furthermore, the evaluation of DCM patients by CMR provides important prognostic information [4,95]. Accordingly, DCM patients have been extensively studied by CMR, and yet CMR data regarding the prognostic value of RV systolic dysfunction in DCM patients are still emerging. Gulati et al. prospectively evaluated 250 DCM patients, demonstrating that patients with RV dysfunction, defined by an RVEF < 45%, had a 4-fold increase in all-cause mortality or cardiac transplantation compared to patients with preserved RV function [4]. Becker et al. evaluated 216 DCM patients by CMR and demonstrated that RV dysfunction was strongly associated with the risk of all-cause mortality and ventricular arrhythmias [96]. Pueschner et al. prospectively evaluated 423 DCM patients, confirming the findings that RV systolic dysfunction is an independent predictor of cardiac mortality, especially in patients with RVEF < 25% (nearly 5-fold higher compared to patients without RV dysfunction) [31]. Consequently, CMR evaluation in DCM patients is clinically relevant, and all DCM patients should be evaluated by CMR at least once [94]. Finally, CMR-derived RV volumes, mass, and EF have high inter- and intra-observer reproducibility, both in patients with normal and dilated RV [4,95]. However, several factors still limit its wide use (i.e., time consuming, expensive, patients with different types of cardiac devices which are CMR-incompatible).

## 5. Conclusions

RV dysfunction is common and yields an unquestionable prognostic significance in DCM patients. In several studies, RV dysfunction emerged as a predictor of adverse events both at baseline and during long-term follow-up. Compared to the classical echocardiographic parameters such as TAPSE, S’ wave velocity, Tei index, and RVFAC, more advanced imaging techniques, namely RV strain by STE, RVEF by 3DE and CMR, and non-invasive echocardiographic surrogates of RVAC, provide improved risk stratification in DCM patients. Multimodality cardiac imaging is of paramount importance for the accurate evaluation of RV systolic function in DCM patients, providing diagnostic, prognostic, and therapeutic implications.

## Figures and Tables

**Table 1:** Advantages, disadvantages, and prognostic significance of the main echocardiographic parameters of right ventricular systolic function.

	Echocardiography	RV Function	Advantages	Disadvantages	Prognostic Value in DCM
TAPSE	- M-mode	- longitudinal - free wall basal segment	- easily obtained - fast - reproducible - wide availability	- angle-dependent - load-dependent - does not evaluate global RV function	- predicts survival and the need for LVAD or cardiac transplantation
S’ wave velocity	- tissue Doppler imaging	- longitudinal - free wall basal segment	- easily obtained - fast - reproducible - wide availability	- angle-dependent - load-dependent - does not evaluate global RV function	- predicts survival
Tei index	- tissue Doppler imaging - pulsed-wave Doppler	- global systolic and diastolic	- easily obtained - fast - wide availability	- less useful in irregular heart rhythms	- not specifically addressed in DCM patients
RV FAC	- two-dimensional	- global	- easily obtained - wide availability - evaluates both longitudinal and radial shortening	- load dependent - poor reproducibility - dependent on image - quality - neglects RV outflow - contribution	- predicts survival and the need for LVAD or cardiac transplantation
RVFWLS	- speckle-tracking	- longitudinal - entire free wall	- less angle- and load-dependent - highly - reproducible - detects subclinical RV dysfunction	- less availability - dependent on image quality - post-processing is necessary	- predicts survival and risk for heart failure decompensation - additive or superior to classical RV parameters
RVEF	- three-dimensional	- global	- does not imply geometrical assumptions - considers the contribution of all RV components - valuable after cardiac surgery - validated against CMR	- limited availability - highly dependent on image quality - post-processing and training are necessary	- predicts survival superior to other RV parameters
RV-PA coupling	- depending on the parameter used	- reflects RV contractility in relation to afterload	- TAPSE/sPAP is validated against RHC-derived parameters - the advantages of respective parameters of RV function apply	- inapplicable in patients without tricuspid regurgitation - the limitations of the respective parameter of RV function apply	- predicts survival and the risk for heart failure hospitalization

Abbreviations: LVAD, left ventricular assist device; RVFWLS, right ventricular free wall longitudinal strain; RV-PA, right ventricular pulmonary artery; other abbreviations as in Figure 1.

## Data Availability

Not applicable.
